# Development of a tetravalent subunit vaccine against dengue virus through a vaccinomics approach

**DOI:** 10.3389/fimmu.2023.1273838

**Published:** 2023-11-17

**Authors:** Amina Basheer, Syed Babar Jamal, Badr Alzahrani, Muhammad Faheem

**Affiliations:** ^1^ Department of Biological Sciences, National University of Medical Sciences, Rawalpindi, Punjab, Pakistan; ^2^ Department of Clinical Laboratory Sciences, College of Applied Medical Sciences, Jouf University, Sakakah, Saudi Arabia; ^3^ Department of Biomedical Sciences, University of North Dakota School of Medicine and Health Sciences, Grand Forks, ND, United States

**Keywords:** immunoinformatics, subunit vaccine, multi-epitope vaccine, proteins, docking, MD simulation

## Abstract

Dengue virus infection (DVI) is a mosquito-borne disease that can lead to serious morbidity and mortality. Dengue fever (DF) is a major public health concern that affects approximately 3.9 billion people each year globally. However, there is no vaccine or drug available to deal with DVI. Dengue virus consists of four distinct serotypes (DENV1-4), each raising a different immunological response. In the present study, we designed a tetravalent subunit multi-epitope vaccine, targeting proteins including the structural protein envelope domain III (EDIII), precursor membrane proteins (prM), and a non-structural protein (NS1) from each serotype by employing an immunoinformatic approach. Only conserved sequences obtained through a multiple sequence alignment were used for epitope mapping to ensure efficacy against all serotypes. The epitopes were shortlisted based on an IC50 value <50, antigenicity, allergenicity, and a toxicity analysis. In the final vaccine construct, overall, 11 B-cell epitopes, 10 HTL epitopes, and 10 CTL epitopes from EDIII, prM, and NS1 proteins targeting all serotypes were selected and joined via KK, AAY, and GGGS linkers, respectively. We incorporated a 45-amino-acid-long B-defensins adjuvant in the final vaccine construct for a better immunogenic response. The vaccine construct has an antigenic score of 0.79 via VaxiJen and is non-toxic and non-allergenic. Our refined vaccine structure has a Ramachandran score of 96.4%. The vaccine has shown stable interaction with TLR3, which has been validated by 50 ns of molecular dynamics (MD) simulation. Our findings propose that a designed multi-epitope vaccine has substantial potential to elicit a strong immune response against all dengue serotypes without causing any adverse effects. Furthermore, the proposed vaccine can be experimentally validated as a probable vaccine, suggesting it may serve as an effective preventative measure against dengue virus infection.

## 1 Introduction

Dengue is a severe life-threatening public health issue in tropical and subtropical regions; more than one-third of the global population is at risk for contracting dengue infection (1). Dengue is a viral infection caused by four antigenically distinct serotypes (DENV 1-4), primarily transmitted by the vector *Aedes aegypti* and secondary vector *Aedes albopictus* (2). Over the last few decades, dengue fever (DF) has emerged as the most common mosquito-borne infection after malaria. There are approximately 50–100 million cases annually, including 250,000 to 500,000 cases of dengue hemorrhagic fever and 24,000 deaths (3, 4). Furthermore, the overall number of dengue fever cases documented by the World Health Organization (WHO) has grown significantly over the past two decades. The number of reported cases has increased by a factor of eight, from 505,430 in 2000 to almost 2.4 million in 2010, and it is anticipated to exceed 4.2 million by 2019. The overall number of recorded deaths has also been risen from 960 in 2000 to 4,032 in 2015. This increase in reported cases underscores the growing impact of dengue fever as a global public health concern. According to these reports and disease modelling estimations, dengue is grossly underreported and the actual burden of the disease is likely much higher than current estimates suggest (5, 6).

The pathogen responsible for dengue fever is known as DENV, an arbovirus belonging to the family Flaviviridae (7). The dengue virus consists of a positive-sense single-stranded RNA genome that encodes a polyprotein that is cleaved by both host and viral proteases into three structural proteins, namely capsid (C), envelope (E), and membrane [prM/M] proteins, and seven non-structural (NS) proteins, NS1, NS2A, NS2B, NS3, NS4A, NS4B, and NS5, during co-translational processing (8, 9). The envelope of DENV is made up of a lipid bilayer that contains two envelope-associated proteins, E and M. The glycoprotein E consists of three domains, namely EDI, EDII, and EDIII (10). The EDIII region of the dengue E protein has been selected as an ideal vaccine target due to its immunodominant properties. In a previous study, four DNA constructs comprising the DIII of a separate DENV serotype were engineered to improve expression and secretion in mammalian cells and evaluated as genetic vaccines in mice. It was found that they induced robust neutralizing responses against all four serotypes (11, 12). Importantly, EDIII has minimal potential for generating cross-reactive antibodies against heterologous dengue serotypes, which are known to be associated with the development of critical disease. Additionally, this domain elicits a type-specific neutralizing response, which has a limited possibility of producing cross-reactive antibodies and possibly alleviating the adverse effects of antibody-dependent enhancement (ADE). Furthermore, the Ig-like EDIII is a stable and independently folding domain, making it an attractive target for recombinant protein expression (13). Another structural prM protein acts as a chaperon of the E protein inside the secretory pathway of the host T cells, preventing the E protein from undergoing premature fusion or inactivation (13). Many studies have shown that anti-prM monoclonal antibodies have been found to significantly improve infection in cell culture and animal models of DENV infection. In a previous study, mice immunized with recombinants that expressed dengue prM and M were protected against dengue encephalitis. It has been demonstrated that prM and M proteins function as antigens that stimulate a protective immune response (14, 15).

Similarly, among the non-structural proteins of the four DENV serotypes, NS1 is the only one released by infected cells and is approximately 79% conserved across all serotypes. During the initial stages of infection, the NS1 protein is secreted into the extracellular space and has a crucial role in viral replication, immune evasion, and disease pathogenesis. The substantial level of conservation of NS1 across all DENV serotypes underscores its importance as a promising target for the development of vaccines (16, 17). Many research studies have demonstrated that NS1 vaccinations are highly immunogenic in mice, provoking a robust humoral and cellular immune response against the NS1 protein. Additionally, antibodies generated against NS1 have been shown to inhibit the harmful effects of released NS1, such as complement activation and cytokine storm (18).

The clinical trials for several different dengue vaccine candidates are ongoing but only one vaccine, Dengvaxia manufactured by Sanofi Pasteur, has been approved by the United States Food and Drug Administration (USFDA). In addition to Dengvaxia, many other dengue vaccines are under development and being evaluated in preclinical studies. The prM and E proteins are the primary focus of vaccine development because they are thought to elicit protective immune responses in humans (19). Furthermore, there are several limitations associated with Dengvaxia: it is relatively less effective against the DENV-2 serotype and has negative consequences for pregnant women, including miscarriage, stillbirth, and foetal death (20). Additionally, Dengvaxia has poor protection efficiency in children under the age of nine. Therefore, it is debatable whether Dengvaxia can be used to prevent DENV infections (21). Considering these circumstances, a new vaccine candidate that offers protection against all serotypes of DENV is imminently required.

All dengue virus serotypes (1–4) can infect humans (22). Despite decades of research, there is still no specific antiviral drug or vaccine that is licensed for human use. As a result, there is an absolute necessity for a dengue vaccine that can prevent us from contracting the viral infection. With the recent advances in bioinformatics, many strategies have been implemented to construct knowledge-based vaccines using the immunoinformatics approach. In the present study, we developed a multi-epitope tetravalent subunit vaccine by employing an immunoinformatics-based approach consisting of highly conserved epitopes of two structural proteins, EDIII and prM, and one non-structural protein (NS1) of DENV1-4 that has the potential to be immunogenic against all four DENV serotypes. Therefore, these epitopes have been selected based on their minimal variation across serotypes, ensuring that the vaccine can target multiple serotypes simultaneously.

## 2 Materials and methods

### 2.1 Retrieval of protein sequences of the DENV 1-4 serotypes

The protein sequences of EDIII, prM, and NS1 of all serotypes (DENV1-4) were downloaded from NCBI GenBank (23), the Virus.Pathogen Resource (ViPR) (24), and UniProt (25). Protein sequences were selected to cover all different regions globally. To find sequence similarity, the reference sequences were subjected to BLASTp (Basic Local Alignment Search Tool) (26) and then integrated into a single FASTA file.

### 2.2 Multiple sequence alignment and conserved domain analysis

The retrieved protein sequences of EDIII, prM, and NS1 were subjected to multiple sequence alignment (MSA) by ClustalW (27) integrated into the BioEdit tool. Consequently, consensus domains of these proteins that were conserved in all dengue virus serotypes were predicted (28).

### 2.3 Epitopes mapping

The linear B-cell epitopes of conserved domains were predicted using the online Immune epitope database and Analysis Resource (IEDB) http://tools.iedb.org/bcell/  (29) with Bepipred Linear Epitope Prediction 2.0 and BcePRED (30). Similarly, CTL epitopes were identified using the prediction method NetMHCpan BA 4.1 http://tools.iedb.org/mhci/, and HTL epitopes were predicted using the prediction method NetMHC-II v.2.3 http://tools.iedb.org/mhcii/. Furthermore, the screening of epitopes was accomplished by sorting the alleles of MHC-I and MHC-II based on their percentile score and IC50 values. The conserved epitopes with an IC50<50nM and a percentile score <10 were selected.

### 2.4 Antigenicity, allergenicity, and toxicity prediction of epitopes

The predicted B and T Cell conserved epitopes were subjected to comprehensive selection criteria, including numerous prediction parameters applied to validate their suitability for addition in the vaccine construct. Furthermore, the parameters that were used for the selection of epitopes included antigenicity, toxicity, and allergenicity. The antigenic score of the epitopes was determined by employing VaxiJen v2.0 (31) at a threshold of 0.47 (available at http://www.ddg-pharmfac.net/vaxijen/VaxiJen/VaxiJen.html), and the allergenic epitopes were predicted by AllerTOP v2.0 (32) (available at https://www.ddg-pharmfac.net/AllerTOP/). To determine the toxicity of the peptides, ToxinPred2 (33) was used for the prediction of toxic epitopes. For the purposes of the subsequent study, only those antigenic, non-allergenic, and non-toxic epitopes were considered.

### 2.5 Molecular docking of HLA alleles with shortlisted T-cell epitopes

The homology modelling of the finalized T-cell epitopes was conducted using the PEP-FOLD3 server (34) to generate the peptide structures. The structures of MHC-I and MHC-II alleles of all epitopes were checked for their availability at the RCSB PDB database (35). The MHC alleles were docked with their respective epitopes using the PatchDock server (36).

The PatchDock molecular docking algorithm was used to identify the docking transformations that lead to favorable shape complementarity between molecules. Only the structure with the minimum optimized potential for efficient structure prediction (sOPEP) energy was selected. For the visualization of interaction, the UCSF Chimera server (37) and BIOVIA Discovery Studio were used to examine the epitopic sites and the basic pattern of amino acids.

### 2.6 *In silico* design of the DENV multi-epitope vaccine, physicochemical properties, and solubility analysis

The multi-epitope subunit vaccine was constructed against all DENV1-4 serotypes by integrating prioritized B- and T-cell epitopes from prM, EDIII, and NS1 proteins. Using a special linker, EAAAK, AAY, and GGGS epitopes were joined together. B-cell, HTL, and CTL epitopes were joined by KK, AAY, and GGGS linkers, respectively. In addition, B-defensins (PDB ID: 1IJV) were incorporated into the vaccine as an adjuvant and positioned at the N termini of epitopes to enhance the immunogenicity of the vaccine. The first epitope was linked to the adjuvant through an EAAAK single linker, and a thrombin site and His tag were employed at the C terminus of the designed vaccine construct.

Furthermore, important physicochemical properties of the designed multi-epitope subunit vaccine were investigated using the ProtParam server’s ExPASy tool (38) The solubility of the multi-epitope vaccine construct was predicted by the Protein-Sol server (http://protein-sol.manchester.ac.uk/).The vaccine was further evaluated for antigenic assesssment by the VaxiJen and AntigenPro tools. Additionally, the vaccine underwent testing on the AllerTOP v.2.0 server to make sure it would not cause any allergic reactions. Collectively, these studies offer insight into the vaccine’s efficacy and safety (32).

### 2.7 Structure prediction of the DENV multi-epitope vaccine

The secondary and tertiary structure of the constructed vaccine was predicted. PSIPRED v.3.3 (39) and PDBsum was used to make predictions regarding the secondary structures of the designed vaccine by employing the primary sequences of the vaccine as input (40). The secondary structure evaluation was used to foresee solvent accessibility, globular area, trans-membrane helix, and turn region, which are crucial factors that contribute to the stability of proteins and the efficacy of vaccines (41).

The tetravalent multiepitope vaccine structure prediction was made by the I-Tasser (42), Phyre2 (43),. and RoseTTAFold servers (44). Furthermore, the structure was refined by GlaxyRefine. The web server GlaxyRefine is used for protein model structure refinement and mainly focuses on improving the quality of local structures (45). A suitable model was selected by evaluating the Rama-favored score and RMSD compared with the initial model. Afterward, the model was confirmed by the ERRAT score and Ramachandran plot analysis. The discontinous epitopes in the three-diensional refined structure were predicted by the ElliPro server (http://tools.iedb.org/ellipro/) (46)

### 2.8 Molecular docking interaction of the vaccine with toll-like receptor 3

The PDB structure of TLR3 was extracted from the RCSB Protein Data Bank (PDB ID:1ZIW). Active and passive residues of the designed construct and TLR3 were achieved using C-PORT (47) before proceeding to docking assessment. Similarly, the HADDOCK server was employed to estimate the interaction of the construct with TLR3 (48). UCSF Chimera and BIOVIA Discovery Studio were used for the visualization of the results.

### 2.9 Codon optimization and *in silico* cloning

The protein sequence of the vaccine construct was reverse translated into nucleotide sequences by EMBOSS Backtranseq (49) followed by codon adaptation. Codon adaptation of the tetravalent vaccine construct was performed using the Java Codon Adaptation Tool (JCat) server (50), and the codons were adapted to be compatible with the most commonly used prokaryotic expression vector, the *E. coli* K12 strain (51). The GC percentage and the CAI were analyzed. SnapGene software (http://snapgene.com/) was used to cloned the improved construct into the pET21(+) vector.

### 2.10 Population coverage analysis

Population coverage prediction is critical for vaccine design to ensure that the designed vaccine construct covers the highest possible global population. The IEDB population coverage tool was used to predict the population coverage of the vaccine construct (available at http://tools.iedb.org/population/) with the prioritized MHC-I and MHC-II epitopes.

### 2.11 Immune simulation analysis

The C-ImmSim server, a web-based dynamic immune simulation tool available at https://kraken.iac.rm.cnr.it/C-IMMSIM/ (52) was used to evaluate an immune response of a designed tetravalent subunit vaccine. The simulation of the vaccine was conducted for a number of time intervals, particularly 1,42,84 with a total of 1,051 simulation steps.

### 2.12 Molecular dynamics simulation

A molecular dynamics (MD) simulation was conducted with a vaccine-docked complex using GROMACS, a Linux-based program (53). The vaccine structure was subjected to simulations to imitate the biological environment encountered by proteins in a biological system. To ensure compatibility with the Optimized Potential for Liquid Simulation-All Atom (OPLS-AA) force field, the vaccine structure was converted into a GRO file, which represents the vaccine’s topology and was ready for use in the MD simulation. After NVT and NPT equilibration at 100 ps, the structure generated was then subjected to 50-ns MD simulations. The root mean square deviation (RMSD) of the structure with the minimized energy predicted and simulation graphs were visualized using QTgrace, and VMD was used to visualize GROMACS files (54).

## 3 Results

### 3.1 Retrieval of protein sequences

The retrieved EDIII, prM, and NS1 protein sequences were subjected to BLASTp using default parameters to obtain the most identical sequences. The reference sequences with their accession numbers retrieved from different regions of the world, including Pakistan, are shown in **Supplementary Table S1**. The number of FASTA protein sequences obtained from different DENV serotypes are shown in **Table 1**.

**Table 1:** DENV serotype FASTA sequences obtained from BLAST.

Protein	DENV1 sequence	DENV2 sequence	DENV3 sequence	DENV4 sequence
EDIII	426	542	484	375
prM	362	239	360	229
NS1	227	459	200	205

Multiple sequence alignment of EDIII, prM, and NS1 proteins of all serotypes revealed the conservation among different serotypes. For downstream analysis, we selected only the conserved protein sequences of each serotype protein (prM, EDIII, and NS1).

### 3.2 Epitope mapping

The epitopes were shortlisted based on different selection criteria, such as IC50, percentile score, and antigenic score. For an epitope to be considered high affinity, the IC50 value should be less than 50 nM. An IC50 value <500 nM implies intermediate affinity, while values <5,000 nM indicate low affinity (55). All epitopes with an antigenic score ≥0.470 and IC50 <50 were selected for vaccine design. We screened a cumulative 76 B-cell epitopes of all three proteins using BepiPred-2.0: Sequential B-Cell Epitope Predictor (56). A total of 44 epitopes of screened B-cell epitopes showed high VaxiJen scores (>0.47), thus showing antigenic characteristics.

The cytotoxic T-lymphocyte (CTL) and helper T-lymphocyte (HTL) epitopes for EDIII for DENV1-4 were predicted using the Immune Epitope Database (IEDB) and were 110 and 117, respectively. Only 28 CTL and 22 HTL epitopes showed good antigenicity scores (>0.47), were non-allergenic and non-toxic, and had IC50 values <50, indicating significant binding potential with the anticipated HLA alleles. Likewise, for prM DENV1-4 serotypes, overall, 39 CTL epitopes and 30 HTL epitopes were predicted; out of these, 21 CTL and 22 HTL epitopes were screened. For NS1 protein DENV1-4 serotypes, overall, 85 CTL and 54 HTL epitopes were predicted, and out of these, 20 CTL epitopes and 28 HTL epitopes were predicted to be potential epitopes and had antigenic scores >0.47, IC50 values <50, and were non-allergenic and non-toxic.

After considering their binding affinities with respect to IC50 values and the highest antigenic score, we shortlisted the best B- and T-cell epitopes from each serotype. To prioritize the epitopes, we chose one epitope of each serotype that had the highest antigenic score and an IC50 <50 nM. In total, 12 B-cell epitopes from EDIII, prM, and NS1 were identified as potential antigenic epitopes. Similarly, a total of 24 T-cell epitopes, including HTL and CTL epitopes, were selected from EDIII, prM, and NS1 from the DENV1-4 serotype to construct a vaccine, as shown in **Tables 2**, **3**.

**Table 2:** Selected B-cell epitopes of EDIII, prM, and NS1.

Protein	Serotype	B-cell epitopes	Antigenic score
EDIII	DENV1	KALKLSW	2.12 (antigenic)
	DENV2	KALKLSWFKKG	1.0088 (antigenic)
	DENV3	TEIQNGGTSIF	0.471 (antigenic)
	DENV4	PIEIRDVNKEK	1.7233 (antigenic)
prM	DENV1	LAPHVGLGLETRTE	1.2893 (antigenic)
	DENV2	PHMIVRQEKGKSL	0.4788 (antigenic)
	DENV3	MCDDTVTYKCPIEVEPEDIDC	0.998 (antigenic)
	DENV4	TRDGEPLMIVKHERGRPLLFKTTEGI	0.6418 (antigenic)
NS1	DENV1	WKGRELK	1.1036 (antigenic)
	DENV2	DGSMSIKNEE	1.6551 (antigenic)
	DENV3	EWCCRS	2.4557 (antigenic)
	DENV4	LKGKRALPPDLK	1.4877 (antigenic)

**Table 3:** Selected CTL and HTL epitopes of the DENV1-4 serotypes.

Protein	Serotype	CTL epitopes	MHC Alleles	IC50	Antigenic Score	Allergenicity	Toxicity	PI	Mol.wt(Da)
**EDIII**	DENV1	KLTLKGSYV	HLA-A*02:03	25.14	1.068(Antigenic)	Non allergen	Non toxin	9.72	1008.36
	DENV2	LTLKGSYVM	HLA-A*68:01	38.18	1.2035(Antigenic)	Non allergen	Non toxin	8.94	1011.38
	DENV3	TILIKVEYK	HLA-A*03:01	38.81	1.7933(Antigenic)	Non allergen	Non toxin	8.83	1106.51
	DENV4	VGSALTLHW	HLA-B*58:01	8.27	1.0554 (Antigenic)	Non allergen	Non toxin	7.1	983.27
**prM**	DENV1	SMAMRCVGI	HLA-A*02:06	39.69	1.5822(Antigenic)	Non allergen	Non toxin	8.6	967.35
	DENV2	RQEKGKSLLF	HLA-B*15:01	**61.97**	0.4506(Antigenic)	Non allergen	Non toxin	10.01	1205.57
	DENV3	LTSRDGEPR	HLA-A*68:01	37.64	1.5125(Antigenic)	Non allergen	Non toxin	6.42	1030.22
	DENV4	LLFKTTEGI	HLA-A*02:03	17.81	0.6686(Antigenic)	Non allergen	Non toxin	6.35	1021.36
**NS1**	DENV1	LSMTCIAVGV	HLA-B*58:01, HLA-A*02:01	21.58	2.366(Antigenic)	Non allergen	Non toxin	5.85	993.39
	DENV2	AAEGINYAD	HLA-A*02:03, HLA-B*35:01	10.45	1.1155 (Antigenic)	Non allergen	Non toxin	3.67	923.06
	DENV3	TAGPWHLGK	HLA-A*11:01	49.93	1.1355(Antigenic)	Non allergen	Non toxin	9.11	966.24
	DENV4	TQTGPWHLGK	HLA-A*31:01	41.23	1.1272 (Antigenic)	Non allergen	Non toxin	9.11	1124.42
Protein	Serotype	HTL epitopes	MHC allele	IC50	Antigenic Score	Allergenicity	Toxicity	PI	Mol.wt(Da)
**EDIII**	DENV1	ALTGATEIQSGTTTI	HLA-DRB1*07:01	34.5	0.7846(Antigenic)	Non allergen	Non toxin	4	1463.8
	DENV2	GSYVMCTGSFKLEKE	HLA-DRB3*01:01	36.8	0.8936(Antigenic)	Non allergen	Non toxin	6.45	1679.15
	DENV3	SNIVIGGDALKINWY	HLA-DRB1*13:02	21.4	0.9058(Antigenic)	Non allergen	Non toxin	9.65	1663.14
	DENV4	EIRDVNKEKVVGRII	HLA-.DRB1*07:01	**79.2**	0.6148(Antigenic)	Non allergen	Non toxin	8.93	1768.32
**prM**	DENV1	LMLVTPSMAMRCVGI	HLA-.DRB1*01:01	39.37	1.0701(Antigenic)	Non allergen	Non toxin	10.11	1666.15
	DENV2	KSLLFKTGNMCTLMA	HLA-DRB1*01:01	51.4	0.47 (Antigenic)	Non allergen	Non toxin	7.09	1632.03
	DENV3	MCDDTVTYK	HLA-DRB1*03:01	1.7	0.77(Antigenic)	Non allergen	Non toxin	8.84	1689.21
	DENV4	DQKAVHADMGYWIES	HLA-DRB1*03:01	20.5	0.6105 (Antigenic)	Non allergen	Non toxin	4.54	1750.13
**NS1**	DENV1	LTWLGLNSRSTSLSM	HLA-DRB3*02:02	31.6	1.9214 (Antigenic)	Non allergen	Non toxin	10.11	1666.15
	DENV2	AAIKDNRAVHADMGY	HLA-DRB5*01:01, HLA-DRB1*15:01	17.24	1.1778 (Antigenic)	Non allergen	Non toxin	7.09	1632.03
	DENV3	GSWKLEKASLIEVKT	HLA-DRB1*01:01	9.4	0.9481(Antigenic)	Non allergen	Non toxin	8.84	1689.21
	DENV4	DQKAVHADMGYWIES	HLA-DRB3*01:01	**69.4**	0.6105 (Antigenic)	Non allergen	Non toxin	4.54	1750.13

*The antigenicity score was provided by VaxiJen. An antigenic score >0.4 indicates that all the epitopes are antigenic in nature. Some of the epitopes we considered in the final vaccine construct are antigenic in nature but have an IC50 >50 (represented in bold). The chosen epitopes are non-allergenic and non-toxic.

**Figure 1:**
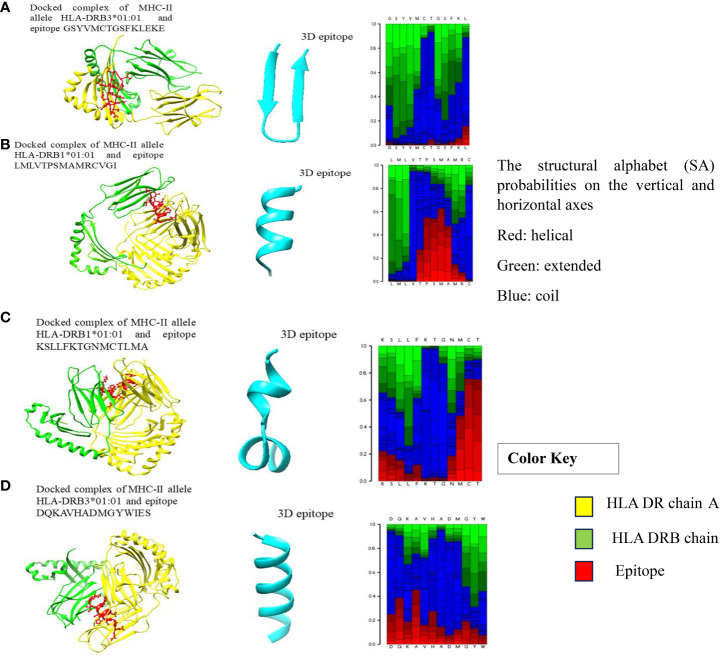
**(A–D)** Docked complexes of MHC-II alleles with their epitopes, showing the structural interactions between the selected HLA epitopes (red), HLA DR chain A (yellow), and HLA DRB chain (green).

Additionally, during the selection process, we observed that the prioritized B-cell epitope of DENV2-EDIII contained KALKLSWFKKG and the smaller fragment of the DENV1-EDIII B-cell epitope, KALKLSW; therefore, the larger epitope was retained from these two epitopes in the final vaccine construct to ensure the best possible outcome (57). Similarly, we also noticed that another DENV1-prM CTL epitope, SMAMRCVGI, consists of a fragment of the DENV1-prM HTL epitope LMLVTPSMAMRCVGI; hence, the larger epitope was considered in a final vaccine construct. Another two epitopes, KLTLKGSYV of DENV1-EDIII CTL and LTLKGSYVM of DENV2-EDIII CTL, exhibited some overlapping residues. Therefore, we merged them to form one continuous epitope, KLTLKGSYVM, in the vaccine construct. In this way, we carefully selected and prioritized the most suitable epitopes for the vaccine construction, as shown in **Figure 1**.

Additionally, in our study, we also considered the DENV2 epitope RQEKGKSLLF associated with HLA-B*15:01 and DEV4 epitope EIRDVNKEKVVGRII associated with allele HLA-DRB1*07:01, despite having slightly higher IC50 values, i.e., 61.97 and 79.2, respectively. They were selected for inclusion in the final vaccine design because of favorable antigenicity scores and binding affinities. These epitopes are represented in bold in **Table 3**. All the selected B-cell, CTL, and HTL epitopes are shown in **Tables 2**, **3**.

### 3.3 Molecular docking of HLA alleles with shortlisted T-cell epitopes

**Figure 2:**
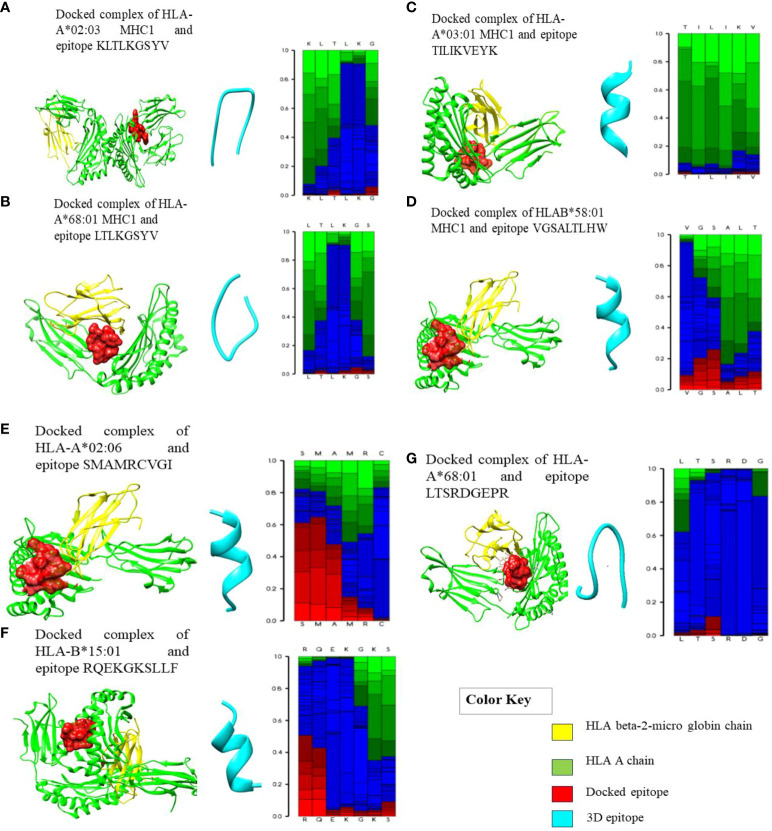
Docked complexes of MHC-I alleles showing interactions with their epitopes as represented above in **(A–G)** illustrating the structural interactions between the selected HLA epitopes (red), HLA A chain (green), and HLA beta-2 micro globin chain (yellow). **(A)** Docked complex of HLA-A*02:03 MHC-I and epitope KLTLKGSYV. **(B)** Docked complex of HLA-A*68:01 MHC-I and epitope LTLKGSYV. **(C)** Docked complex of HLA-A*03:01 MHC-I and epitope TILIKVEYK. **(D)** Docked complex of HLAB*58:01 MHC-I and epitope VGSALTLHW. **(E)** Docked complex of HLA-A*02:06 and epitope SMAMRCVGI. **(F)** Docked complex of HLA-B*15:01 and epitope RQEKGKSLLF. **(G)** Docked complex of HLA-A*68:01 and epitope LTSRDGEPR.

Molecular dockings were carried out between the modelled epitope and their putatively interacting HLA alleles. This was essential to perform structural analysis of the interaction between the epitope and the HLA alleles. Furthermore, the docking of epitopes with HLA molecules is the first stage in triggering specific immune responses. During the development of vaccines, determining the epitopes that have a statistically significant binding affinity for HLAs should be prioritized for inclusion in vaccine formulations. Epitopes are presented on the surface of antigen-presenting cells (APCs), such as dendritic cells; when they interact with HLA molecules, the resulting interaction triggers a series of immunological responses that ultimately leads to the generation of immune cells that are particular to the antigen (57) The structures of MHC-I and MHC-II alleles of all epitopes were checked for their availability in the RCSB PDB database, and some of the alleles that have no associated crystal structures are available in the RCSB PDB database. Therefore, we selected only those alleles with PDB structures for docking. Notably, the MHC-II epitopes GSYVMCTGSFKLEKE, LMLVTPSMAMRCVGI, KSLLFKTGNMCTLMA, and DQKAVHADMGYWIES showed striking binding energies with HLA-DRB3*01:01, HLA-DRB1*01:01, HLA-DRB1*01:01, and HLA-DRB3*01:01, respectively. The best MHC-II protein-epitope docked structures are shown in **Figure 1**. These figures also showed that stable binding can be achieved through strong peptide-protein interactions within the HLA molecule’s binding groove. The MHC-I alleles were docked with their respective epitopes using the PatchDock server and their docked complexes are shown in **Figure 2**. Alleles with their PDB IDs are presented in **Table 4**.

**Table 4:** HLA’s interacting epitopes with PDB IDs.

HLAs	HLA PDB ID	Interacting epitope
HLA-A*02:03	3OX8	KLTLKGSYV (DENV1-EDIII CTL)
HLA-A*68:01	6PBH	LTLKGSYVM (DENV2-EDIII CTL)
HLA-A*03:01	7L1C	TILIKVEYK (DENV3-EDIII CTL)
HLA-B*58:01	5IND	VGSALTLHW (DENV4-EDIII CTL)
HLA-A*02:06	3OXR	SMAMRCVGI (DENV1-prM CTL)
HLA-B*15:01	6UZP	RQEKGKSLLF (DENV2-prM CTL)
HLA-A*68:01	6PBH	LTSRDGEPR (DENV3-prM CTL)
HLA-A*02:03	3OX8	LLFKTTEGI (DENV4-prM CTL)
HLA-A*02:01	7M8S	LSMTCIAVGV (DENV1-NS1 CTL)
HLA-A*68:01	6PBH	AAEGINYAD (DENV2-NS1 CTL)
HLA-A*11:01	7OW4	TAGPWHLGK (DENV3-NS1 CTL)
HLA-DRB3*01:01	2q6W	GSYVMCTGSFKLEKE (DENV2 -EDIII HTL)
HLA-DRB1*01:01	1H15	LMLVTPSMAMRCVGI (DENV1-prM HTL)
HLA-DRB1*01:01	IBX2	KSLLFKTGNMCTLMA (DENV2-prM HTL)
HLA-DRB3*01:01	2q6W	DQKAVHADMGYWIES (DENV4-NS1 HTL)

The chosen T-cell epitopes had strong interactions with their corresponding HLA alleles and the PDB structure with the lowest ACE score (atomic contact energy) was selected. The best HLA protein-epitope docked complexes of some epitopes with their respective alleles are visualized in **Figure 2**. These graphical representations represent the strong protein-epitope bonding within the binding region of the HLA molecule, which is necessary for maintaining a stable interaction. Furthermore, after docking to verify the binding groove of HLAs with their respective epitopes (also checked on the PDB database), it was observed that the epitopes were interacting with the same HLA chain.

### 3.4 The design (*in silico*) of multi-epitope vaccine construct

**Figure 3:**
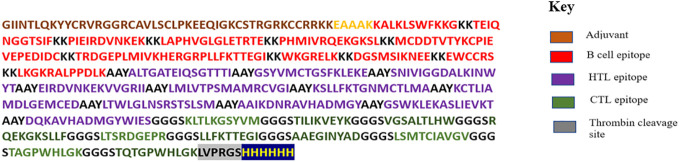
The complete sequence of amino acids included in the vaccine construct. B-defensin adjuvant is added to the construct at the N terminus. Specific linkers (EAAAK, AAY, KK, and GGGS) were included to distinguish selected epitopes.

The vaccine construct was developed by incorporating the screened epitopes based on the criteria discussed above. In total, 11 B-cell epitopes, 10 helper T-cell (HTL).epitopes, and 10 cytotoxic T-cell (CTL) epitopes were chosen from DEN1-4 (the proteins EDIII, prM, and NS1) and joined using KK, AAY, and GGGS linkers, respectively. Following the completion of the merging process, it was determined that the total length of the construct was 500 amino acids. A 45-amino acid-long adjuvant (GIINTLQKYYCRVRGGRCAVLSCLPKEEQIGKCSTRGRKCCRRKK) was added at the N terminus of the construct by the EAAAK linker. Subsequent to the incorporation of linkers in addition to the adjuvant, a thrombin cleavage site and 6×histidine tag were inserted at the C terminus. The final vaccine construct was determined to be 557 amino acids long. The sequence of the final vaccine construct formulated is shown in **Figure 3**.

### 3.5 Property analysis of the designed vaccine protein

A physicochemical study revealed that the molecular weight of the vaccine construct is 60.23 kDa. It is important to note that proteins with molecular weights of less than 110 kDa have a greater chance of exhibiting antigenic characteristics (58). The *in vitro* half-life of the vaccine was identified as 30 h in mammalian reticulocytes, whereas *in vivo* it was 20 h in yeast and 10h in *Escherichia coli*. The protein instability index was 29 (<40), which indicates a high probability of stability. The aliphatic index was 75.03; a higher aliphatic index value of proteins indicates thermostability, even at extreme temperatures. Moreover, a GRAVY (grand average of hydropathicity) score of -0.379 was obtained; a negative GRAVY value shows the hydrophilic characteristics of proteins (59). The solubility of the vaccine construct was predicted to be 0.6 by Protein-Sol. For the experimental dataset (PopAvrSol), the population average was 0.45; hence, any scaled solubility value more than 0.45 is expected to have higher solubility than the average soluble *E. coli* protein (60).

Based on these findings, it is anticipated that the multi-epitope vaccine satisfies the physicochemical feature requirements for further analysis. The antigenic score of the designed vaccine was found to be 0.7968 after being evaluated on both the AntigenPro and VaxiJen servers; the threshold against the virus target organism was 0.4, suggesting that the designed vaccine is probable antigenic. Similarly, the AllergenFP and AllerTOP analysis for allergenicity proposed that the vaccine had non-allergic features, making it improbable that it would cause allergic reactions in humans, and ToxinPred analysis predicted that the vaccine construct was non-toxic (61).

### 3.6 Population coverage of vaccine construct

**Figure 4:**
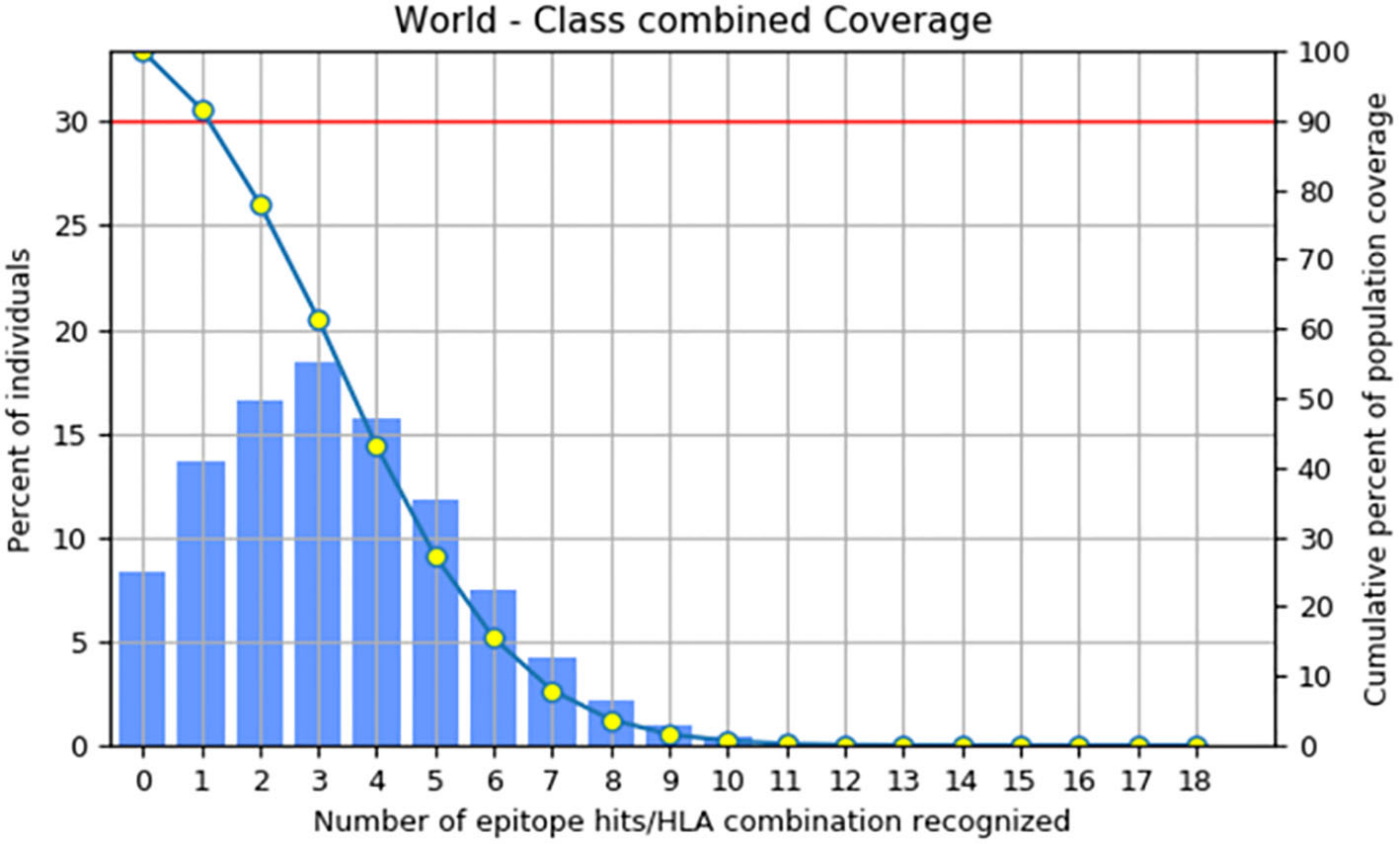
Coverage of the combined MHC-1 and MHC-II epitopes across the world’s population.

The primary objective of population coverage assessment is to determine the suitability of the candidates for large populations (62). The population coverage of the construct was estimated to be 91.67%, as shown in **Figure 4** by the IEDB population coverage online tool. The worldwide distribution of the constructed vaccine provided more evidence that it was successful for the vast majority of the world’s population.

### 3.7 The prediction of secondary and tertiary structures and validation

**Figure 5:**
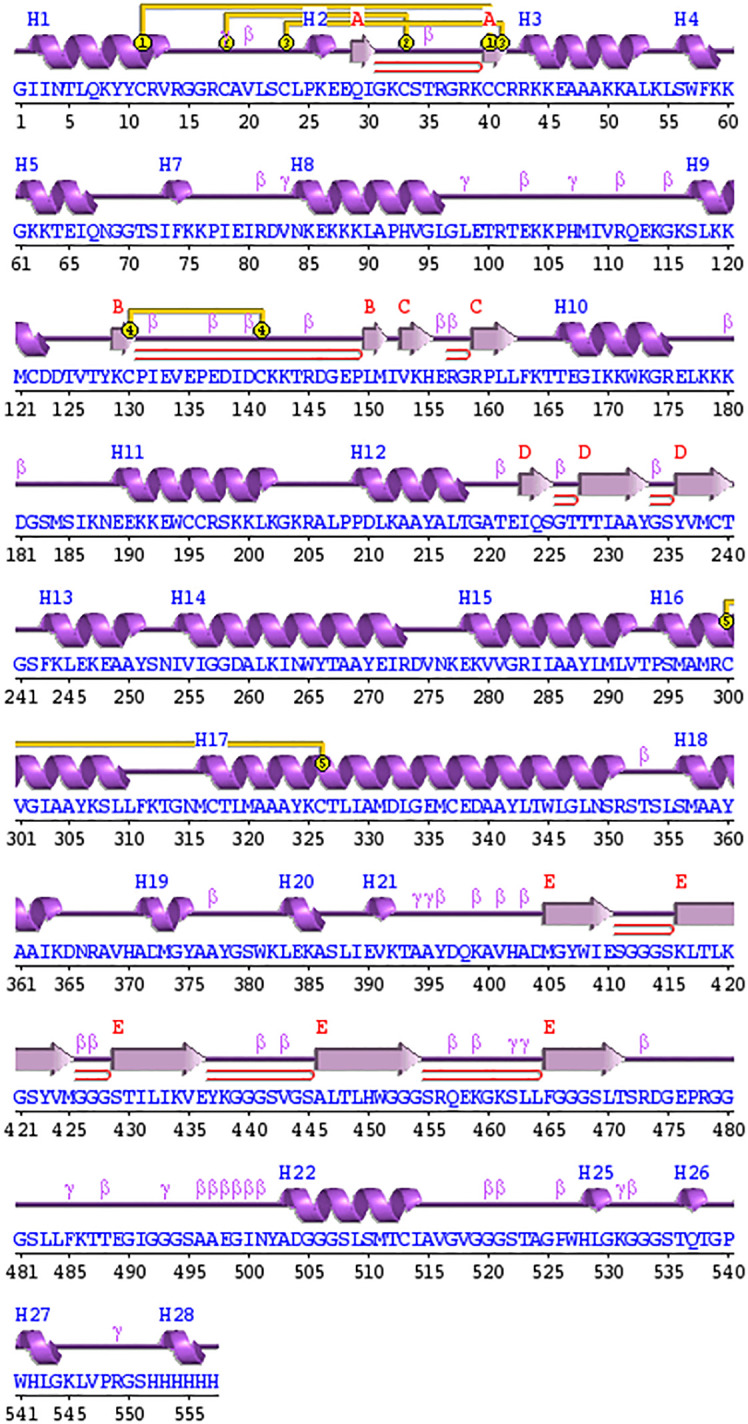
Schematic depiction of the secondary structure obtained for the final tetravalent subunit vaccine. The ⍺-helices have been labelled as H and β-sheets are marked as A, B, C, D, and E. The red loops show the b-hairpins, and disulphide bonds are represented by the yellow joining bar.

The secondary structure of the designed vaccine construct was predicted by PDBsum and revealed that the structures are composed of 28 alpha helices, 5 β sheets, 9 beta hairpins, and 5 disulphide bonds, as shown in **Figure 5**. Additionally, when the secondary structure was compared with the tertiary structure, it was found that the number of alpha and β-sheets were homologous, which increases the confidence level of the predicted structure.

**Figure 6:**
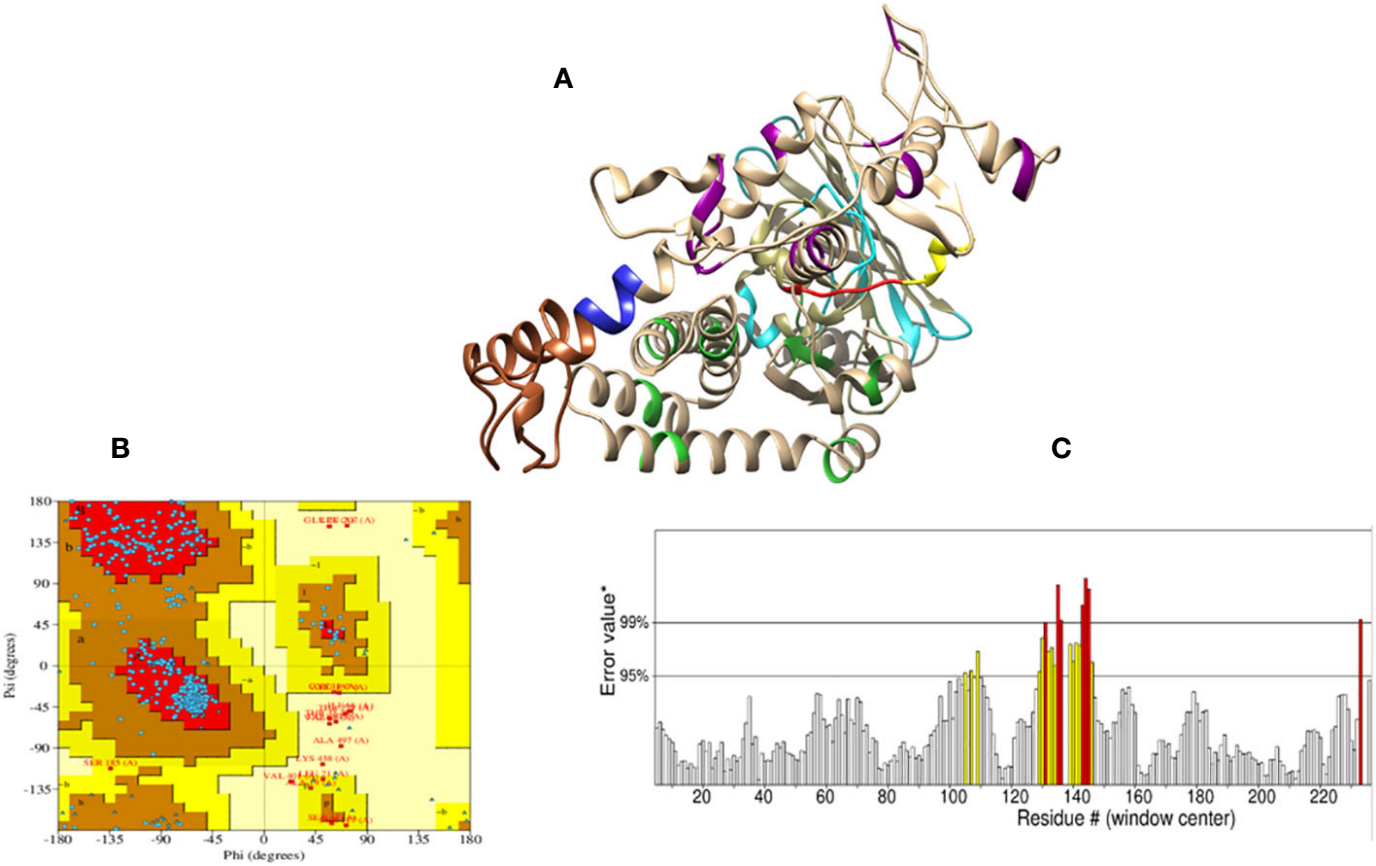
Prediction model of the vaccine construct. **(A)** The tertiary structure of the vaccine construct. The model shows the His-tag (yellow), adjuvant (brown), thrombin site (red), EAAAK linker (blue), KK linker (dark magenta), AAY linker (lime green), and GGGS linker (cyan); light brown represents all the epitopes present in the vaccine construct. **(B)** Ramachandran plot analysis for the quality assessment of the model revealed that 84.4% of the residues are present and the most favored regions; 12% of the residues are present in additionally allowed regions. **(C)** A stereochemical evaluation performed by ERRAT showed a higher ERRAT value of 93.84, which indicates a highly refined structure. The X-axis represents the amino acid residue number (#) in a protein model and y axis represents the error value*. On the y-axis, labelled “Error value*”, two distinct lines have been plotted to show the level of confidence in which we can determine the areas that have error values greater than the stated threshold. In some cases, residues with error values of greater than 99% were found; these regions are shown as red. The regions with error values greater than 95% are shown as yellow.

Initially, the tertiary structure of the vaccine construct was predicted by I-TASSER, PHYRE2, and RoseTTAFold; the RoseTTAFold web server produced better results, as shown in **Figure 6A**. The RoseTTAFold web server generated five models; of these, one model was chosen based on the highest Ramachandran and ERRAT scores. Moreover, the molecular refinement by GlaxyRefine did not improve the generally Rama-favored score of the vaccine model. Hence, we selected model 05 with a higher Rama-favored score for further analysis. The refinement scores of all models are shown in **Supplementary Table S2**. Furthermore, Ramachandran plot statistics showed that 84.4% of residues were located in the furthermost favored regions and 12% of residues were present in allowed regions; hence, the overall score was predicted to be 96.4% (**Figure 6B**), and the ERRAT score was 93.84, which shows the high quality of a tertiary structure. The ERRAT plot is shown in **Figure 6C**. The ElliPro server predicted six discontinous B-cell epitopes in three-dimensional structure, with scores between 0.504 and 0.81. The range of epitope sizes comprised 3-163 residues, as represented in **Supplementary Table S3**.

### 3.8 Molecular docking analysis of the vaccine construct with TLR-3 receptors

Interaction with the immune receptor (TLRs) is necessary for the intracellular transport of an antigen molecule and the activation of the proper downstream immune pathways. In this regard, we conducted docking of a multi-epitope tetravalent vaccine construct with TLR3 to assess the intermolecular interactions and binding energy (61).

**Figure 7:**
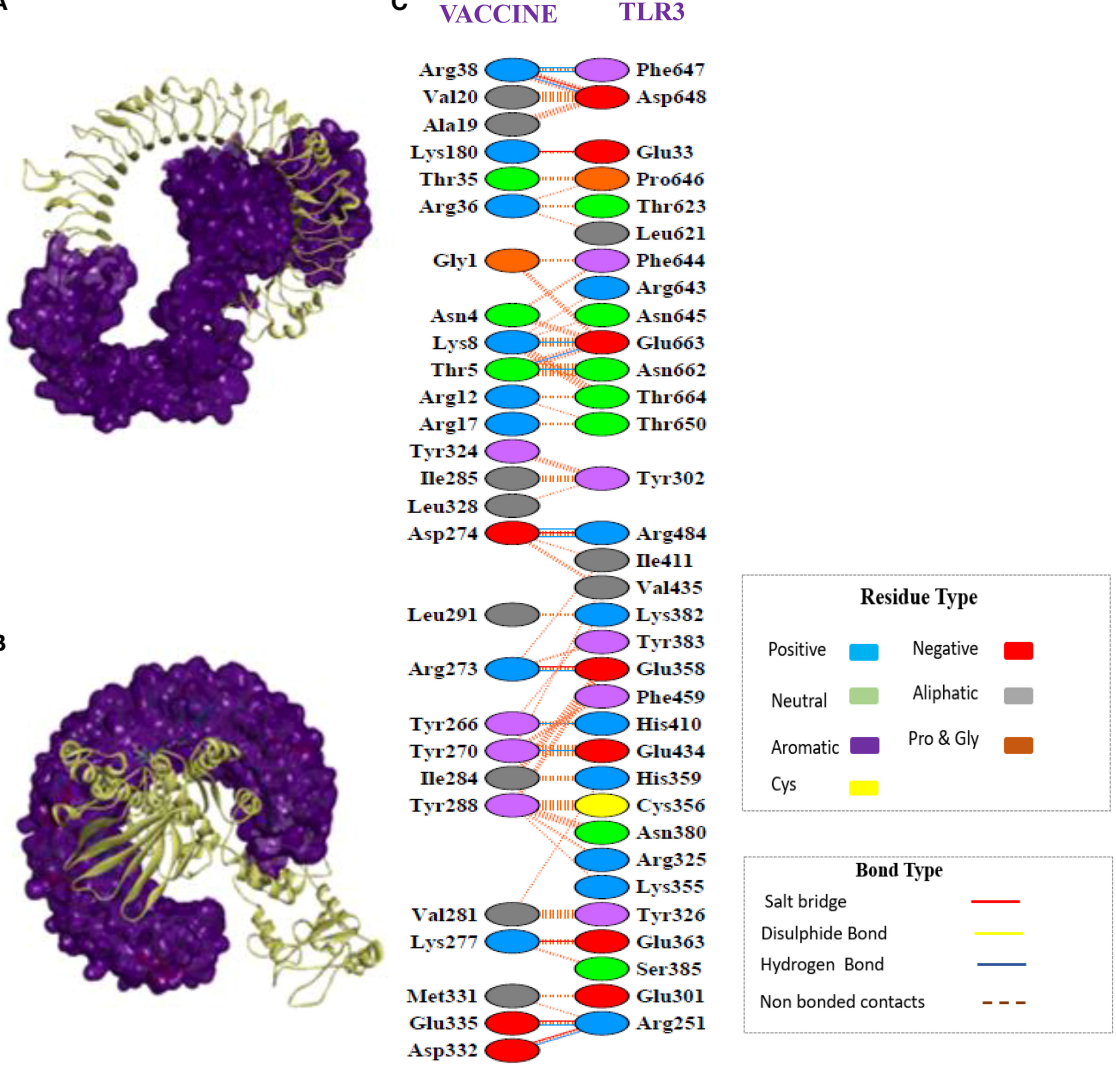
Protein-protein docking interaction of the vaccine and TLR3. **(A)** Docked complex of the multi-epitope vaccine with TLR3. Here, the structure of the vaccine is presented in ribbons, whereas TLR-3 is represented in surface form. **(B)** The same complex as in **(A)**; however, the TLR3 structure is presented in the form of ribbons and the vaccine structure is represented in surface form. **(C)** Secondary structure analysis showed many non-bonded contacts, which are represented as dotted lines between the vaccine and TLR3, and some important hydrogen bonds are indicate by blue lines and salt bridges are indicated by red lines. Additionally, the types of amino acid residues that participated in the interactions were determined, namely aliphatic, aromatic, positive, and negative.

The CPORT analysis before docking showed that the vaccine construct has active residues at positions 1, 2, 3, 4, 5, 6, 9, 10, 12, 13, 34, 262, 263, 266, 267, 270, 271, 273, 274, 281, 282, 284, 285, 288, 291, 292, 293, 294, and 324, and TLR-3 active residues are present at positions 322, 323, 353, 355, 356, 379, 380, 614, 616, 618, 634, 638, 639, 643, 644, 647, 652, 653, 654, 655, 656, 657, 658, 659, 660, 661, 662, 663, 664. Similarly, the HADDOCK server revealed a docking score of –108.02+/–6.4 for the docked complex. A docked complex of vaccine-TLR3 is visualized in **Figures 7A, B**, which illustrates how tightly the putative vaccine binds within the receptor-binding region. The interface contacts between the vaccine construct and TLR3 complex revealed the presence of significant intermolecular interactions. These interactions included hydrogen bonding, non-bonding, and salt-bridge interactions, which are shown in **Figure 7C**.

### 3.9 Codon optimization and *in silico* cloning

**Figure 8:**
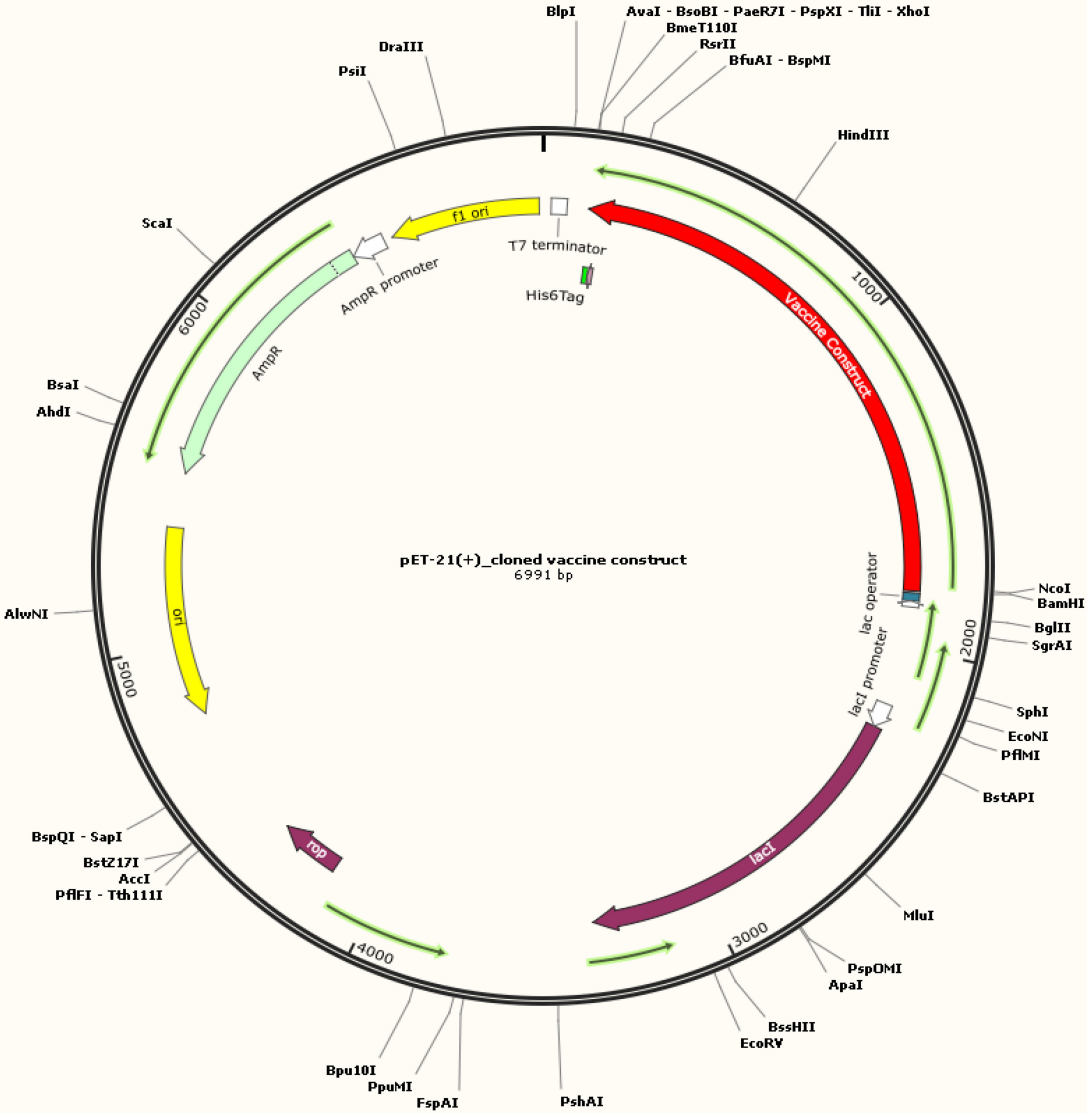
*In silico* restriction cloning of the codon-optimized multi-epitope subunit vaccine construct in the pET-21(+) vector between restriction sites (*XhoI* and *BamH1*). *E. coli* strain K12 can be used to express the final designed construct for vaccine manufacturing. The black circle shows the vector, and the red area denotes the vaccine construct.

*In silico* cloning was conducted to validate the expression of recombinant vaccine protein using an *E. coli*-K12 model (63). The genetic code (gene) was deduced from the protein structure and the codons were optimized. The optimized gene had 1,671 nucleotides, GC content was predicted to be 48.77% (the ideal range is between 30% and 70%), and the CAI value was 1.0 (the ideal range is 0.8–1.0), which can be employed to determine protein expression (64). At the N and C termini of the optimized construct, appropriate restriction sites, *XhoI* and *BamH1*, respectively, were included. The tetravalent construct was then cloned across these sites of vector pET21 (+) at multiple cloning sites. Thus, the complete clone was 6,991 base pairs in length (**Figure 8**).

### 3.10 Immune simulation analysis

**Figure 9:**
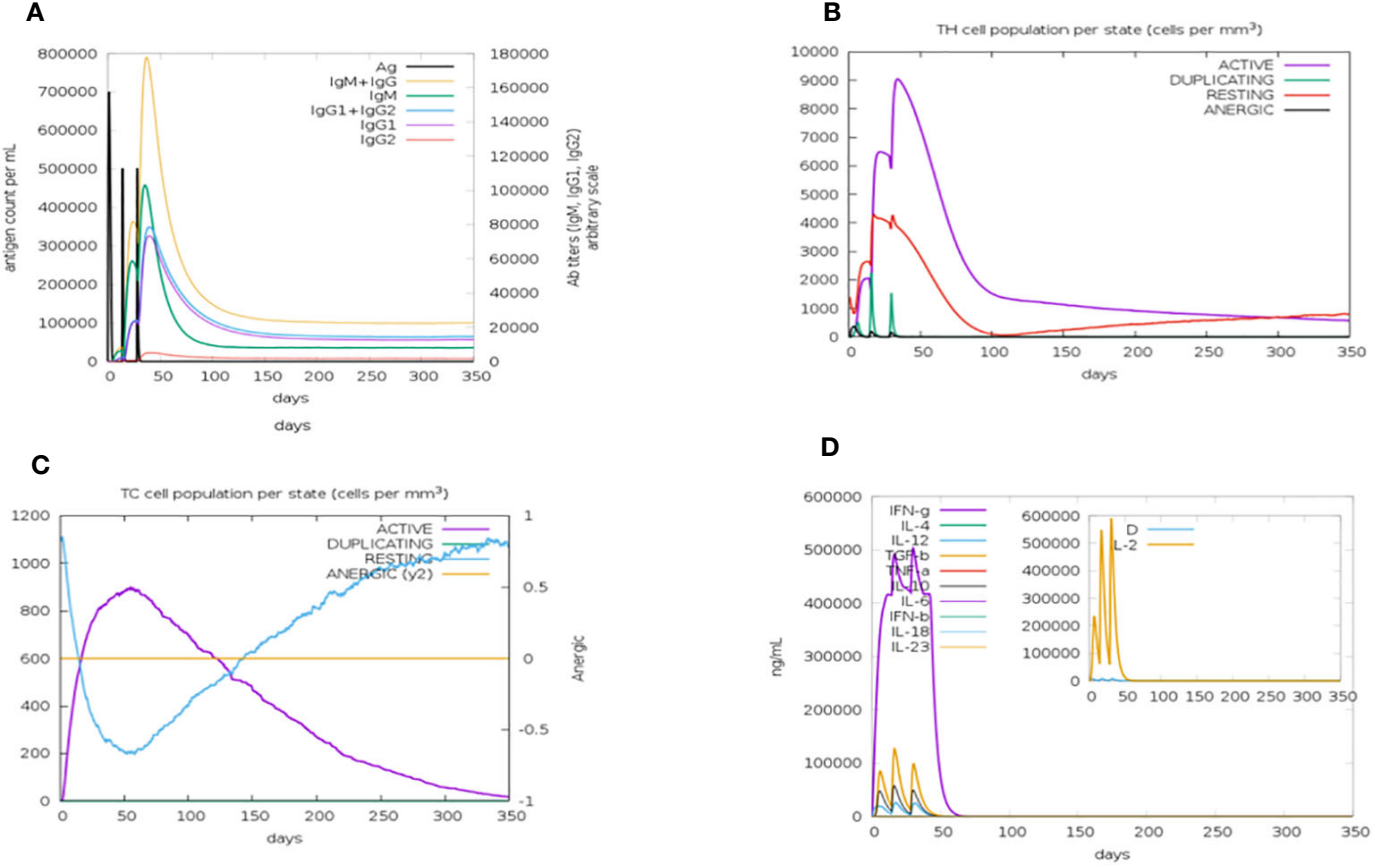
The immune simulation results of the designed vaccine construct after three injections of time intervals 1,42,84 using a C—immSim server at simulation step 1050. **(A)** An increase in the production of immunoglobulins in response to antigen injection. **(B)** The total number of TH cells. Active T-helper (TH) cells with a peak of the relative count of 9,000 cells per mm3 between 10 and 40 days. **(C)** The total number of TC lymphocytes in all states (active, duplicating, resting, and anergic). The number of active TC cells reached a peak of more than 900 cells per mm3 after 30 days following chimeric vaccine administration. **(D)** Cytokine and interleukin production graph. The levels of IFN- (more than 500,000 ng/ml) and a higher Simpson index represented by D of IL-2 in the graph indicate the production of several cytokines as a response to the multi-epitope vaccine antigen.

The C-ImmSim server was used to simulate how the body’s immune system would react to the designed vaccine. According to immune simulation results, our designed vaccine elicits robust cellular and humoral immune responses. When an antigen is encountered for the first time, the body develops a primary immune reaction, and in most cases, IgM is the predominant antibody produced, with a small quantity of IgG also generated. The primary immune response, in the form of IgM antibodies (>140,000), substantially increased after the first injection of the vaccine construct (antigen). The secondary immune reaction is characterized by elevated levels of IgM and IgG (>160,000) and develops in response to repeated exposure to the same antigen. Aditionally, there was a notable rise in the concentration of IgM and IgG and a fall in antigen concentration. IgM, IgG1, and IgG2 levels also increased significantly, as shown in **Figure 9A**. After the injections, there were higher amounts of both active T-helper cells and T-cytotoxic cells in the populations of each state. Significantly, helper T lymphocytes remained at a higher population level throughout the exposure. The anergic phase is represented by an ability to tolerate T cells upon antigen exposure; the resting state denotes the cells that were not subjected to antigens, as depicted in **Figures 9B and C**. Similarly, a higher IFN concentration score compared with other cytokines was observed (**Figure 9D**).

### 3.11 MD simulation analysis

**Figure 10:**
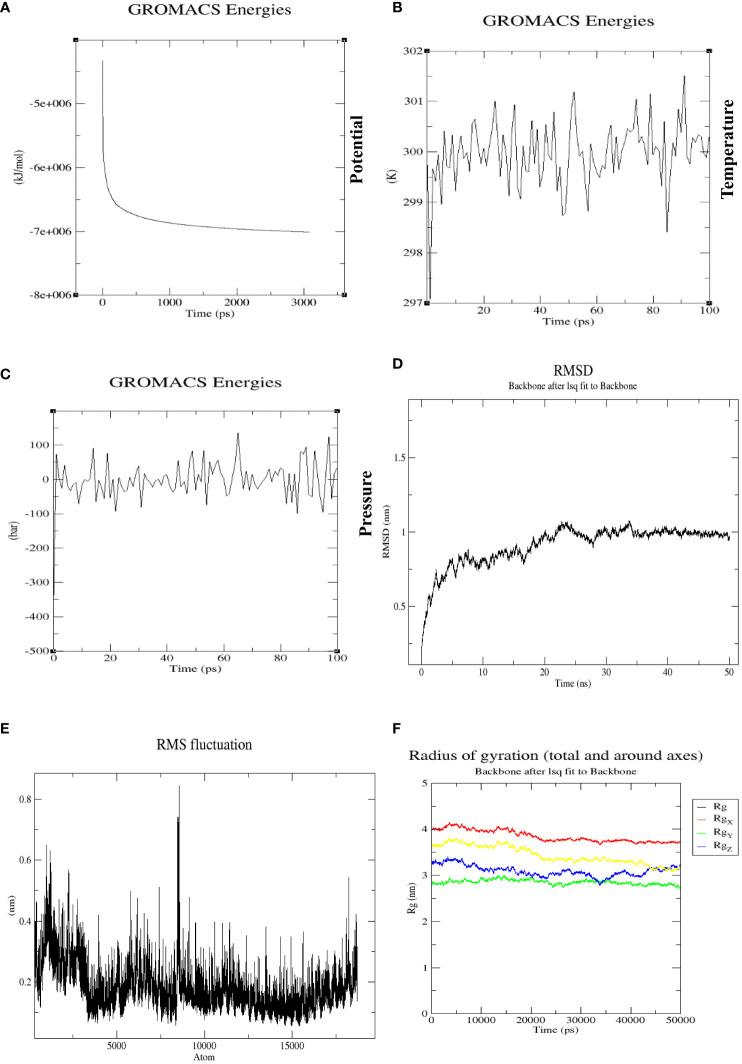
**(A)** The potential energy plot during the simulation at 3,000 ps was found to be −7e+006, which shows that our system has the minimum energy to start real dynamics. **(B)** Average temperature progression plot of the vaccine-TLR3 complex, which remained at 299.77K over 100 ps. **(C)** Average pressure plot fluctuations, which remained at 1 bar during a 100-ps equilibration. **(D)** RMSD plot of vaccine-TLR3 complex over a 50-ns MD production run. There was a noticeable rise in the RMSD for this system until 35 ns (beginning at 0.5 nm and reaching a peak at an RMSD of 1 nm), with slight fluctuations. After that, the system eventually reached a stable state and maintained an RMSD of 0.9 nm, which represents the stability of complex. **(E)** The RMSF of a complex during MD production simulation. The residues in the designed vaccine-TLR3 complex were evaluated for flexibility using RMSF. The highest fluctuating residues were observed in atoms between 5,500 and 9,000. **(F)** Rg of the complex around all axes with minimum fluctuations, indicating the compactness of the vaccine-TLR3 complex during the whole MD simulation run of 50 ns.

The molecular dynamic simulation was conducted on docked complexes using GROMACS 5.0 software to verify the stability of contacts between the multi-epitope tetravalent vaccine complex and the receptor TLR-3. Energy minimization was performed for 50,000 steps to remove clashes, and equilibration was conducted to bring the system to the desirable conditions in terms of temperature, pressure, density, etc. The energy minimization process was completed at 3,000 ps, as represented in **Figure 10A**. The temperature was stable at 299.7K for a period of approximately 100 ps, as shown in **Figure 10B**, and remained stable throughout the equilibration phase, whereas pressure varied, with a mean value of 1 bar during the same period of 100 ps, as represented in **Figure 10C**. Similarly, the backbone RMSD plot in **Figure 10D** shows that the protein structure did not equilibrate more rapidly, and at the beginning of the plot, RMSD was 0.5–0.75 nm at 10 ns. In this plot, RMSD fluctuates between 20–25 ns up to 1 nm and then maintains RMSD after 35 ns, which shows that the complex is stable throughout the simulation run. The root means square fluctuation (RMSF) plot of the protein exhibited distinct peaks, indicative of pronounced conformational variability observed in a multitude of residues within the vaccine-TLR3 complex shown in **Figure 10E**. The stability and compactness of the docked complex were calculated using the radius of gyration (Rg), which showed that all the positions were compact, with the complex around all axes shown in **Figure 10F**. This plot indicates that the Rg of the complex is stable during the whole simulation without too many fluctuations.

## 4 Discussion

Dengue virus (DENV) is an arbovirus belonging to the family *Flaviviridae*, consisting of four serotypes (DENV1-4) that cause DF, dengue shock syndrome, and dengue hemorrhagic fever (65). It is estimated that DENV is responsible for more than 390 million infections every year, and in excess of 96 million cases are recorded with disease manifestations (66). Although DENV is a fatal virus, there is no approved drug or vaccine available that is equally effective against all the serotypes.

Dengvaxia, developed by Sanofi-Pasteur, has been the only licensed vaccine against DENV infection. Dengvaxia varied in its protection among the DENV serotypes, demonstrating a protective efficiency of 50.3% against DENV1, 42.3% against DENV2, 74.0% against DENV3, and 77.74% against DENV4. Additionally, Dengvaxia has poor protection efficiency in children under the age of nine. Therefore, it is debatable whether Dengvaxia can be used to prevent DENV infection (21). The development of conventional vaccines is progressing but the co-circulation of multiple serotypes and antibody-dependent enhancement processes are restricting progress (67).

The present study is based on employing an immunoinformatics approach, considering potential vaccine candidate proteins, including EDIII, prM, and non-structural protein NS1, to design tetravalent subunit vaccines. The immunogencity of these proteins has been already tested in previous experimental studies and promising results against dengue virus infections were obtained (11–15, 18). In recent years, the field of immunoinformatics has undergone significant advancements that have introduced a plethora of reliable tools and servers for vaccine development. These tools have revolutionized the process of vaccine development, resulting in significantly reduced time and cost requirements compared with traditional methods. We have adopted an immunoinformatics approach and screened candidate epitopes in EDIII, prM, and NS1proteins that were substantially conserved between all DENV serotypes. This approach not only covers a large population across the world but is also effective against all of its serotypes because of its high conservancy. The B- and T-cell epitopes were prioritized based on their antigenic score, physicochemical parameters, allergenicity score, and toxicity. These selected epitopes have an ideal binding affinity to MHC molecules and TLR3, and their IC50 values are less than 50 nM. The Toll-like receptors (TLRs) are critical for pathogen recognition and the production of cytokines in several viruses. TLR3 plays a critical role in the activation of natural immune responses against viruses. TLR3-mediated signaling is one of the most important response pathways to type I IFN production following viral dsRNA structure exposure, and it can induce pro-inflammatory cytokines. Although both TLR3 and TLR7 are necessary to produce a type I IFN response in reaction to a DENV infection, TLR3 is more effective than TLR7 in both the induction of IFN and the inhibition of DENV (68, 69). Tsai YT et al. (69) investigated the interaction between DENV and TLRs *in vitro*. The findings revealed that when TLR3 is expressed in HEK293 cells, they induce the secretion of IL-8 in response to DENV recognition. In human monocytic cells (U937), the primary mechanism triggering IL-8 production after viral identification involves TLR3 following endosomal acidification. Silencing TLR3 in U937 cells results in a notable reduction in DENV-induced IL-8 production. One of the most critical requirements for a vaccine is that it establishes strong interactions with immune receptors to facilitate its optimal absorption and distribution across the host body. The results of molecular docking of the vaccine with TLR3 demonstrate that only a minimal amount of energy is needed to create a stable complex with strong interactions between the vaccine and TLR3. These findings suggest that our designed vaccine can potentially stably bind to immune receptors (66). A stable docked complex with good interactions between the vaccine construct and TLR3 signifies that our vaccine can potentially stimulate a robust immune response. TLR3 is crucial in initiating innate immune responses, and if a designed vaccine construct can bind to TLR3 efficiently, it can enhance the activation of immune cells. Its interaction with TLR3 is a positive indicator that it is recognized as a probable pathogen, thereby triggering a more specific immune response.

In addition, we used molecular docking to investigate the structural correlations between these shortlisted T-cell epitopes and their corresponding HLA allele(s). We accomplished this by employing the PatchDock server, a powerful and efficient program for protein-peptide binding, which showed a robust correlation within epitopes and their associated human leukocyte allele(s). Accordingly, a tetravalent vaccine was constructed by joining the prioritized B-cell, HTL, and CTL epitopes via linkers along with the β-defensin adjuvant. The β-defensins as an adjuvant work as an antimicrobial and immunomodulatory agent. Defensin peptides help the host defend itself against microbial infection and play a role in adaptive immunity by attracting naive T cells and immature dendritic cells to the place of infection, thereby boosting antiviral immunity. Similarly, the linkers contribute significantly to extend conformation (flexibility), the folding of proteins, and the separation of functional domains. As a result, the protein structure is rendered more stable because of the contributions of the linkers (70). The HTL epitopes were linked by AYY, which helps the epitopes to generate suitable sites for binding to the TAP transporter and improves epitope presentation (71), and GGGS linkers were added to provide additional flexibility to that region. The presence of GLY residues guarantees that the linker region will be flexible and will not fold into secondary structures (72). To maintain the capacity for independent immunogenic activity, B-cell epitopes were combined with the assistance of KK linkers.

The antigenicity score of the multi-epitope construct was predicted to be 0.7968 by the online tool VaxiJen, with a molecular weight of 60.23 kDa. Furthermore, it is generally believed that proteins with a molecular weight of less than 110 kDa are considered more antigenic in nature. In a three-dimensional refined model of the vaccine, six discontinous B-cell epitopes were identified. Our proposed vaccine can generate substantial antibody formation. This is because discontinuous B-cell epitopes serve an important role in humoral immune responses by generating antibodies (73). However, administration of the multi-epitope vaccine construct does not result in inflammation or allergic reactions. The results of a molecular docking study showed that the anticipated construct forms a strong and stable connection with the TLR3 receptor at a relatively low binding energy (74). According to the immune simulation findings, the vaccine produced a robust number of antibodies, particularly IgM, IgG, T-helper cells, and cytotoxic T cells. Furthermore, MD simulation analysis by GROMACS at 50 ns also showed our vaccine-TLR3 complex is stable throughout the MD production run. Moreover, the implementation of codon adaptation and *in silico* cloning techniques assure enhanced expression of the designed vaccine in the *E. coli* expression system. Based on these findings, we conclude that the tetravalent subunit vaccine we anticipated has a high binding affinity with immune receptors and satisfactory computational evaluation.

## 5 Conclusion

Over the past few decades, dengue virus has spread rapidly across the world, becoming a major public health problem in tropical and subtropical regions, and millions of new cases are recorded. By employing an immunoinformatics approach, we designed a multi-epitope-based tetravalent vaccine targeting DENV1-4 serotypes using the conserved sequences of the proteins EDIII, prM, and NS1. The resulting vaccine construct was further investigated for allergenicity, antigenicity, and toxicity and underwent an immune simulation analysis. All of these analyses predicted that our designed vaccine is highly immunogenic, non-allergenic, and non-toxic. In addition, immune stimulation results demonstrated that both antibody and cell-mediated immune responses would be elicited against DENV by injecting the multi-epitope vaccine. Molecular docking and MD simulation were carried out to validate the interactions within the vaccine-TLR3 complex; the results showed that it exhibited a higher binding affinity and stable interaction. Our results propose that a designed multi-epitope vaccine has significant potential in provoking a balanced immune response against all dengue serotypes without causing any undesirable effects. Moreover, the designed vaccine can be further experimentally validated before human immunization to validate its efficacy and safety profile against dengue virus infections.

## Data availability statement

The original contributions presented in the study are included in the article/**Supplementary Material**, further inquiries can be directed to the corresponding author/s.

## Author contributions

AB: Data curation, Methodology, Software, Writing – original draft, Writing – review & editing. SJ: Conceptualization, Funding acquisition, Project administration, Resources, Supervision, Writing – review & editing. BA: Formal Analysis, Writing – review & editing. MF: Conceptualization, Funding acquisition, Resources, Supervision, Writing – review & editing.
