# Supplementary material for: Development of a tetravalent subunit vaccine against dengue virus through a vaccinomics approach
Source: Front Immunol. 2023 Nov 17;14:1273838. doi: 10.3389/fimmu.2023.1273838 (PMC10690774; doi:10.3389/fimmu.2023.1273838)
Supplement: Supplementary file 1 [file Table_1.docx]

**Development of a Tetravalent Subunit Vaccine against Dengue Virus through Vaccinomics Approach**

Amina Basheer^1^, Syed Babar Jamal^1^, Badr Alzahrani^2^, Muhammad Faheem^1,3,^*

^1^ Department of Biological Sciences, National University of Medical Sciences, Rawalpindi, Punjab, 46000, Pakistan Rawalpindi [aminabasheer205@gmail.com](mailto:aminabasheer205@gmail.com) ; [ba bar.jamal@numspak.edu.pk](mailto:ba%20%20bar.jamal@numspak.edu.pk)

^2^ Department of Clinical Laboratory Sciences, College of Applied Medical Sciences, Jouf University, Sakaka, Saudi Arabia baalzahrani@ju.edu.sa

^3^ Department of Biomedical Sciences, University of North Dakota School of Medicine & Health Sciences, Grand Forks, ND 58202, USA [muhammad.faheem@und.edu](mailto:muhammad.faheem@und.edu)

*****Correspondence: [muhammad.faheem@und.edu](mailto:muhammad.faheem@und.edu)

Supplementary Material

Table S1: DENV serotypes across different regions of the world

|  | DENV Envelope Domain (EDIII) | | | |
| --- | --- | --- | --- | --- |
|  | **Protein** | **Viral serotype** | **Accession number** | **Region** |
|  | EDIII | DENV-1 EDIII | ABK54369.1 | China |
|  |  |  | ABG75766.1 | Hawaii |
|  |  |  | P33478.2 | Singapore |
|  |  |  | 6DFJ\|E | United States |
|  |  |  | 5VIC\|E | Nauru/West Pac |
|  |  |  | ALG00119.1 | Pakistan |
|  |  |  | ALG00128.1 | Pakistan |
|  |  | DENV-2 EDIII | BCG29765.1 | Japan |
|  |  |  | BBH51307.1 | Bangladesh |
|  |  |  | P29991.1 | PDK53 |
|  |  |  | P30026.1 | China |
|  |  |  | P27914.1 | Tonga |
|  |  |  | 2JSF_A | Peru |
|  |  |  | 3J8D | Thailand |
|  |  |  | AHC72406.1 | Pakistan |
|  |  |  | AFP56203.1 | Pakistan |
|  |  |  | AIU39217.1 | Pakistan |
|  |  |  | P07564.2 | Jamaica |
|  |  |  | P14338.1 | Malaysia |
|  |  | DENV-3 EDIII | AMQ36111.1 | France |
|  |  |  | BAM28865.1 | Japan |
|  |  |  | QEP41653.1 | Mumbai,India |
|  |  |  | UBI73841.1 | India |
|  |  |  | QTE05719.1 | Yangon, Myanmar |
|  |  |  | Q99D35.1 | China |
|  |  |  | Q5UB51.1 | Singapore |
|  |  |  | AHC72431 | Pakistan |
|  |  |  | AHC72430 | Pakistan |
|  |  |  | AHC72427 | Pakistan |
|  |  | DENV-4 EDIII | NP_740317.1 | Maryland |
|  |  |  | BCG29769.1 | Japan |
|  |  |  | AEV66314.1 | Vietnam |
|  |  |  | P09866.2 | Dominica |
|  |  |  | Q5UCB8.1 | Singapore |
|  |  |  | AHC72432.1 | Pakistan |
| DENV prM | | | | |
|  | **Protein** | **Viral serotype** | **Accession number** | **Region** |
|  | prM | DENV-1 prM | ABO21759.1 | East Timor |
|  |  |  | AAL67810.1 | Mexico |
|  |  |  | NP_733807.2 | Bethesda, Maryland |
|  |  |  | NP_722459.2 | Bethesda, Maryland |
|  |  |  | P27909.2 | Brazil |
|  |  |  | P27913.1 | Jamaica |
|  |  | DENV-2 prM | AAL67814.1 | Mexico |
|  |  |  | ARO34432.1 | Central America |
|  |  |  | ATQ48916.1 | India |
|  |  |  | P30026.1 | China |
|  |  |  | QZZ92461.1 | Saudi Arabia |
|  |  |  | AGV28546.1 | Pakistan |
|  |  | DENV-3 prM | AAL67821.1 | Mexico |
|  |  |  | AFN80339.1 | Australia |
|  |  |  | AAU29556.1 | Mozambique |
|  |  |  | AVW83051.1 | Pakistan |
|  |  | DENV-4 prM | QXF68706.1 | Eastern Uttar pradesh, India |
|  |  |  | AAL67829.1 | Mexico |
|  |  |  | Q5UCB8.1 | Singapore |
| DENV NS1 | | | | |
|  | **Protein** | **Viral Serotype** | **Ref Accession no** | **Region** |
|  | NS1 | DENV-1 NS1 | P27909.2 | Brazil |
|  |  |  | P17763.2 | Nauru/West Pac/1974 |
|  |  |  | CAO01554.1 | Saudi Arabia:Jeddah |
|  |  |  | BBG62286.1 | northern Vietnam |
|  |  |  | QHE24281.1 | Philippines |
|  |  |  | AKK23333.1 | China |
|  |  | DENV-2 NS1 | P07564.2 | Jamaica |
|  |  |  | CAA78918.1 | Australia |
|  |  |  | AAD11533.1 | Japan |
|  |  |  | AAK67712.1 | Australia |
|  |  |  | AHC72409.1 | Pakistan |
|  |  |  | AIU39216.1 | Pakistan |
|  |  |  | AAA66406.1 | USA |
|  |  | DENV-3 NS1 | AGW99229.1 | India |
|  |  |  | AAZ94620.1 | South America |
|  |  |  | AHC72428.1 | Pakistan |
|  |  |  | UBI73841.1 | India |
|  |  | DENV-4 NS1 | AHC72432 | Pakistan |
|  |  |  | NP_740318 | Maryland 21205, USA |
|  |  |  | AUF73704.1 | China |
|  |  |  | APT69957.1 | Brazil |
|  |  |  | AUF73704.1 | China |
|  |  |  | BAJ72475.1 | Philippines |

**Table S2: Refinement analysis of vaccine construct**

| Model | GDT-HA | RMSD | MolPobity | Rama Favored |
| --- | --- | --- | --- | --- |
| Initial model | 1 | 0 | 1.672 | 91.9 |
| MODEL-1 | 0.9753 | 0.344 | 2.036 | 93.3 |
| MODEL-2 | 0.9771 | 0.342 | 2.148 | 93.9 |
| MODEL-3 | 0.966 | 0.364 | 2.01 | 93.3 |
| MODEL-4 | 0.9654 | 0.373 | 2.01 | 93.3 |
| MODEL-5 | 0.9632 | 0.389 | 2.117 | 94.4 |

**Table S3 Predicted discontinuous epitopes**

| No. | Residues | Number of residues | Score |
| --- | --- | --- | --- |
| 1 | A:G1, A:I2, A:I3, A:N4, A:T5, A:L6, A:Q7, A:K8, A:Y9, A:Y10, A:C11, A:R12, A:V13, A:R14, A:G15, A:G16, A:R17, A:C18, A:A19, A:V20, A:L21, A:S22, A:C23, A:L24, A:P25, A:K26, A:E27, A:E28, A:Q29, A:I30, A:G31, A:K32, A:C33, A:S34, A:T35, A:R36, A:G37, A:R38, A:K39, A:C40, A:C41, A:R42, A:R43, A:K44, A:K45, A:E46, A:A47, A:A48, A:A49, A:K50, A:K51, A:A52, A:L53, A:L55, A:S56, A:W57, A:F58, A:K59, A:K60, A:G61, A:K62, A:K63, A:T64, A:E65, A:I66, A:Q67, A:N68, A:G69, A:G70, A:T71, A:S72, A:I73, A:F74, A:K75, A:K76, A:P77, A:I78, A:E79, A:I80, A:R81, A:D82, A:V83, A:N84, A:K85, A:E86, A:K87, A:K88, A:K89, A:L90, A:A91, A:P92, A:H93, A:V94, A:G95, A:L96, A:G97, A:L98, A:E99, A:T100, A:R101, A:T102, A:E103, A:K104, A:K105, A:P106, A:H107, A:M108, A:I109, A:V110, A:R111, A:Q112, A:E113, A:K114, A:G115, A:K116, A:S117, A:L118, A:K119, A:K120, A:M121, A:C122, A:D123, A:D124, A:T125, A:V126, A:T127, A:Y128, A:K129, A:C130, A:P131, A:I132, A:E133, A:V134, A:E135, A:P136, A:E137, A:D138, A:I139, A:D140, A:C141, A:K142, A:K143, A:T144, A:G147, A:E148, A:P149, A:L150, A:M151, A:I152, A:V153, A:K154, A:H155, A:E156, A:R157, A:G158, A:R159, A:P160, A:L161, A:F163, A:K164, A:T165, A:T166, A:E167 | 163 | 0.81 |
| 2 | A:Y266, A:T267, A:A269, A:Y270, A:E271, A:I272, A:R273, A:D274, A:V275, A:N276, A:K277, A:E278, A:K279, A:V280, A:V281, A:G282, A:I284, A:I285, A:A287, A:Y288, A:L291, A:V292, A:T293 | 23 | 0.657 |
| 3 | A:Y342, A:W345, A:L346, A:G347, A:L348, A:N349, A:S350, A:S352, A:T353, A:S354, A:L355, A:S356, A:M357, A:A358, A:A359, A:Y360, A:A361, A:A362, A:I363, A:K364, A:D365, A:N366, A:R367, A:A368, A:V369, A:H370, A:A371, A:S380, A:W381, A:K382, A:L383, A:E384, A:K385, A:A386, A:S387, A:L388, A:I389, A:E390, A:V391, A:K392, A:T393, A:A394, A:A395, A:Y396, A:D397, A:Q398, A:K399, A:A400, A:V401, A:H402, A:A403, A:D404, A:W408, A:E410, A:S411, A:G412, A:G413, A:G414, A:S415, A:K416, A:L417, A:T418, A:K420, A:G421, A:S422, A:E436, A:Y437, A:K438, A:G439, A:G440, A:G441, A:S442, A:V443, A:G444, A:S445, A:T471, A:S472, A:R473, A:D474, A:G475, A:E476 | 81 | 0.629 |
| 4 | A:V424, A:M425, A:G426, A:G427, A:G428 | 5 | 0.62 |
| 5 | A:K247, A:A250, A:Y251, A:S252, A:N253 | 5 | 0.535 |
| 6 | A:E335, A:E338, A:D339 | 3 | 0.504 |
